# Origin of the Diphtheretic Virus

**Published:** 1889-04

**Authors:** 

**Affiliations:** Frankfort, O. M.


### ORIGIN OF THE DIPHTHERETIC VIRUS.

BY DR. DEICHLER, FRANKFORT, O. M.

The increasing prevalence of diphtheria and the constant struggle carried on against it, too often without result, call the attention of practical physicians anew and anew to the originating causes of this pernicious disease.

Not possessed of the necessary time for experimental studies, he finds himself compelled to draw conclusions from his observations at the sick-bed, to form a theory which, at best, may deserve probation by experiment.

Numerous efforts to discover a specific micro-organism of the diphtheretic virus have failed, until now, to meet with indubitable success, while septic infections of animals by inoculation of diphtheretic mucous matters had to be considered as mixed infections, caused either by schizomycetæ, present in the normal oral fluid and pathogenic for various animals, or attributable to adventitious micro-organisms, originating with the local morbid process.

From *Heubner's* beautiful researches we have learned that the formation of a *Bretonneau* skin may be induced by artificial disturbance in the alimentation of a mucous membrane and consequent necrotic coagulation in *Weigart's* meaning, without immediate co-operation of bacteria. It is true that, in the disease, the diphtheretic membrane on the mucosa of the pharyngeal cavity develops under conditions entirely different from those of the skin artificially produced in the rabbit bladder by disturbance of circulation. But the possibility, and even probability, that a similar primary necrosis by coagulation of the mucous membrane may, in pharyngeal diphtheria, obtain by mechanical or chemical lesion without original bacterial co-operation, is certainly not to be contested

Primary diphtheria may be caused without contagion by some miasma, but diphtheria, originating in this way, is contagious.

Here, we are confronted by the question, if a specific pathogenic germ of the diphtheric membrane is not demonstrable, what is the cause of diphtheria produced without contagion, by some miasma? and further on, where reside the contagium, attached to diphtheric matters which have their origin in a miasma?

Primary pharyngeal diphtheria, originating, as it were, autochthonically, without contagion, is next to *Bretonneau's* skin, produced artificially. But an arrest of blood-circulation, as used by *Heubner* for the purpose of producing a necrosis of the mucous membrane, can not be supposed in this case. It might be said that, through prolonged influence of cold, especially after preceding heat, stagnations in the sanguineous and lymphatic circulations of the throat will set in and favor effusions of rapid coagulation on and into the mucous membrane. But if such every day influences alone were able to produce a diphtheric membrane, we would have met with numberless cases of diphtheria long ago. Our present mode of living and dressing is not so radically different from that prevailing about three decades ago, that to it alone could be ascribed the causa movens of this thoroughly modern disease. We have to admit that the noxious agent producing the primary *Bretonneaus* skin contains a substance which, in former times, when diphtheria appeared in single cases only, existed in small quantity and is being produced now, through changed conditions of life, in larger quantities and spread by means of the atmospheric air. Bacterial action apparently being excluded in the formation of primary diphtheria, not caused by contagion, this substance could not belong to the class of ptomaina, but from its effects it is to be concluded that it is easily propagated by the air and that it is possessed of corrosive properties. A substance, possessing these latter properties in the highest degree and having the faculty of exciting diphtheric processes, is ammonia. It would have to be demonstrated that ammonia is now-a-days present in the atmosphere in larger quantities than before.

In this respect, I wish to call attention to one fact. One of the most striking differences between the habits prevailing in our times and those of former times is the enormous increase in the combustion of coal. Ammonia and its combinations, especially cyanic combinations, are an important product of combustion of coal, particularly of bituminous and rapidly burning machine coal. The poisonous, volatile, empyreumatic products, accompanying the combustion, are whirled into the air together with the smoke of numerous chimneys. Attached to the carbonic particles of smoke, they are carried away by winds of long range, and drift into the pharyngeal cavity of a person, possibly very far away. There, especially when catarrhal irritation is already in existence, they meet with an easy deposit on the tonsilac which frequently present asperities and disruptions. The corrosive properties of ammonia enter into action; according to the quantity of carbonic particles inhaled, the more or less numerous grayish-white spots are formed, which, when in close proximity, coalesce and may, dependent on their number and more or less energetic action, cause a process of coagulatory necrosis on the mucous membrane.

Ammonia certainly answers all requisites of a virus, producing the primary diphtheric membrane of the pharyngeal cavity. In order to be spread throughout the air as a volatile substance, without losing its corrosive properties, it needs some solid body as a conductor. This property is possessed by the carbonic particles of smoke and they are excellent conductors for the purpose mentioned. The predisposition, observed in certain persons, especially children, to have their pharyngeal cavity affected by diphtheria, does not seem solely to depend on existing catarrhal conditions. I am inclined to consider as well founded, the observation, confirmed by professional associates, that primary pharyngeal diphtheria preferently attacks children of delicate features and narrow lips, having a tendency to keep the oral cavity open; it may easily be imagined that such children are less protected from the introduction of particles of smoke than those possessing thick, bulging lips which afford a much tighter closing of the oral cavity.

Thus, the origin of the membranes of local diphtheria, not caused by contagion, may be explained by the action of a chemical, not organized, substance, penetrating from outside and not from the blood. But how is it with regard to secondary pharyngeal diphtheria, especially scarlatinous diphtheria? In this case, there is a general infection before formation of the membrane, and yet, the diphtheric phenomena are not to be distinguished from those of primary pharyngeal diphtheria. Nor is it admissible to suppose a deep seated alimentary disturbance of the pharyngeal membrane, induced by scarlatinous infection and causing a coagulatory necrosis, as provoked on the mucous membrane of the bladder of a rabbit in *Heubner's* experiment; for why should the scarlatinous virus preferably select the vessels of the pharyngeal mucosa, to destroy their functional ability? Schizomycetæ of specific action were not demonstrable in scarlatinous diphtheria, no more than in primary pharyngeal diphtheria, originating without contagion; there, too, the usual parasites, present in the oral mucosities, alone were found, accompanied by some other species, owing their origin to the local morbid process and endowed with the property of giving rise to septic phenomena, but without engendering affections of a specifically diphtheric nature. *Heubner* is certainly right in concluding that a micro-organism, entitled to the name of an indubitable procreator of local and general diphtheric affection, ought to be demonstrated, not only in the mucous matters of the overlayers, but above all in the blood. But in infections, produced in animals by inoculation of mucous matters, their blood and other organs never contained any schizomycetæ besides those known as generators of septic affections.

It would seem, therefore, that in scarlatinous diphtheria as well, the virus, originating in the membranes, consists of a chemical substance, acting corrosively, not generated by schizomycetæ, and inducing the same local process as the virus of primary diphtheria. In both cases, analgous in their symptoms, the first origin of the membrane might be traced to the same identical virus, the only difference being that, in scarlatinous diphtheria, this virus is not introduced from outside, but by the sanguiferous vessels. We have to suppose that, in the scarlatinous affection, the chemically acting substance, contained in the humors, reaches the surface of the mucous membrane through the numerous glands of the pharynx and of the tonsils, admixed to their secretion, there to be decomposed in intimate contact with the atmospheric air and the highly permeatal salivary fluids, and to adopt corrosive properties.

Here, urea will first occur to our mind. The disturbance of the renal function, so frequent in scarlatina, and no less the diminution of perspiration, will favor in an unusual degree an accumulation of urea in the general humors. We know that urea, when its secretion is impeded, will be found, in large quantities, in the secretions of other organs, in which respect I only mention the accumulation of urea on the outer skin in so-called cholera-uraemia. It is to be added that in children, who constitute the chief contingent of scarlatinous diphtheria, in consequence of more active exchange, more urea is secreted than in adult persons.

No less in favor of such a supposition is the easy decomposition of urea in ammonical combinations. If in the putrefaction of urine through the fermentative action of micro-organisms, playing their part in the case, urea is transformed into carbonate of ammonia, it will meet in the pharyngeal cavity with similar conditions, even in a higher degree. There, not only air, heat, humidity, numerous micro-organisms will favor its decomposition, it enters into direct contact with a chemical ferment, ptyaline.

An objection might be raised against this proposition: if corrosion of the mucous membrane through ammonia is to generate scarlatinous pharyngeal diphtheria, why then in the pharyngeal cavity alone, and not simultaneously in the mucous membrane of the oral cavity, this latter membrane being equally provided with numerous glands? This objection will be sufficiently answered by remembering that the smooth surfaces of the oral cavity, together with the motions of the tongue, will offer greater obstacles to the formation of adhering membranes than the generally uneven tonsils which present numerous points of attachment and are, moreover, devoid of any considerable locomotion. There, the membranes may easily establish themselves and furnish a soil, on which the numerous schizomycetæ, living in the oral cavity, may multiply, adopt pathogenic properties and not only assist in spreading the diphtheric process in the pharyngeal cavity, but acquire the quality of actual diphtheric virus.

Primary diphtheria as well as scarlatinous diphtheria can be produced by a chemically acting miasma, without immediate co-operation of bacteria; but diphtheria, produced in this way, is contagious. The existence of specific micro-organisms, as agents of this contagion, has not been demonstrated. On the other hand, we observe, in the diphtheric process, numerous schizomycetæ, living also in the normal oral mucosity, which, having been transferred to new conditions of alimentations, have adopted new properties. We find them in the pharyngeal cavity as well, as in the blood and in the organs of animals, septically infected by inoculation with these diphtheric masses. The conclusion may be allowed that these micro-organisms, having become pathogenic, are to be considered the medium of propagation of contagious diphtheric virus.—*Deutsche Medizinal-Zeitung.*

